# A day in the life of chromatin: how enhancer–promoter loops shape daily behavior

**DOI:** 10.1101/gad.314187.118

**Published:** 2018-03-01

**Authors:** Benjamin J. Weidemann, Kathryn Moynihan Ramsey, Joseph Bass

**Affiliations:** Department of Medicine, Feinberg School of Medicine, Northwestern University, Chicago, Illinois 60611, USA

**Keywords:** circadian rhythms, chromatin topology, promoter–enhancer loops, DNA regulatory elements, transcriptional bursting

## Abstract

Each spring, we get out of bed 1 h ahead of our biological wake-up time due to the misalignment of internal clocks with the light–dark cycle. Genetic discoveries revealed that clock genes encode transcription factors that are expressed throughout many tissues, yet a gap has remained in understanding the temporal dynamics of transcription. Two groups now apply circular chromosome conformation capture and high-throughput sequencing to dissect how “time of day”-dependent changes in chromatin drive core clock oscillations. A surprise is the finding that disruption of enhancer–promoter contacts within chromatin leads to an advance in the “wake-up” time of mice. Furthermore, the assembly of transcriptionally active domains of chromatin requires the ordered recruitment of core clock transcription factors each day. These studies show that waking up involves highly dynamic changes in the three-dimensional positioning of genes within the cell.

## Abstract (precis)

This Outlook by Weidemann et al. highlights two recent studies, one of which, by Mermet et al., is in this issue of *Genes & Development*. They examine how “time of day”-dependent changes in chromatin drive core clock oscillations and show that waking up involves highly dynamic changes in the three dimensional positioning of genes within the cell.

Circadian clock genes, among the first ones identified to control behavior, are encoded by an autoregulatory feedback loop in which transcription factors in the forward limb activate their own repressors in the negative limb, generating oscillations with a periodicity of ∼24 h (Hardin et al. 1990). The discovery that the core clock is also widely expressed outside of the central nervous system in animals led to recognition that cell-intrinsic clocks in turn drive the oscillation of a vast number of genes, enabling anticipation of daily changes in the external environment imposed by the light–dark cycle. Circadian processes are also subjected to post-transcriptional and post-translational regulation, although, at its core, gene regulatory mechanisms remain central to understanding what makes a clock “tick.” Genomic approaches have identified distinct phases in clock transcription factor binding, histone modifications, and RNA polymerase II recruitment to DNA over the 24-h time scale (Koike et al. 2012), with a surprise being that rhythmic gene regulation occurs within enhancer regions that are far away in linear sequence from the proximate transcription start site of oscillating targets (Fang et al. 2014). A question has therefore arisen: How might interactions between regulatory enhancers and oscillating genes contribute to anticipatory behavior and physiology? To address this challenge, both the work in this issue of *Genes & Development* by Naef and colleagues (Mermet et al. 2018) and studies by Lazar and colleagues (Kim et al. 2018) exploit newly available tools from biochemistry and genomics to probe how chromatin reconfiguration evolves over the course of just 1 d. Especially surprising is the finding by Mermet et al. (2018) that genetic abrogation of a specific intronic enhancer element discovered through conformational analyses of chromatin alters rhythmic locomotor activity, suggesting that such loops are necessary for the maintenance of a behavior. Likewise, Kim et al. (2018) provide an unbiased survey of enhancer–promoter interactions, pinpointing a role of the repressor REV-ERBα as a regulator of the clock repressor Cryptochrome 1 (CRY1) and highlighting its function as a core component of the clock.

Just as discovery of the clock provided evidence for temporal control of gene transcription, insight into spatial control across the genome has advanced with the advent of next-generation sequencing. To this end, both the Naef group (Mermet et al. 2018) and the Lazar group (Kim et al. 2018) apply variations of chromatin analyses based on “3C” (chromatin conformation capture) approaches (Fig. 1). The basic strategy is to cross-link chromatin-bound DNA and then identify the pairing of distant regulatory sequences through sequential steps of restriction endonuclease digestion followed by ligation, reverse cross-linking, and sequencing of the ligated fragments (for in-depth discussion, see Denker and de Laat 2016). The key to this powerful technology is the concept that nucleotides within enhancers widely separated in linear sequence from the promoters that they target may occupy proximate spatial topologies due to enhancer–promoter looping. Such enhancer–promoter looping can be frozen upon cross-linking (because, by definition, they are in proximity in three-dimensional space), thereby enabling subsequent ligation and sequence-based identification of topologically colocalized regulatory sequences.

**Figure 1.**
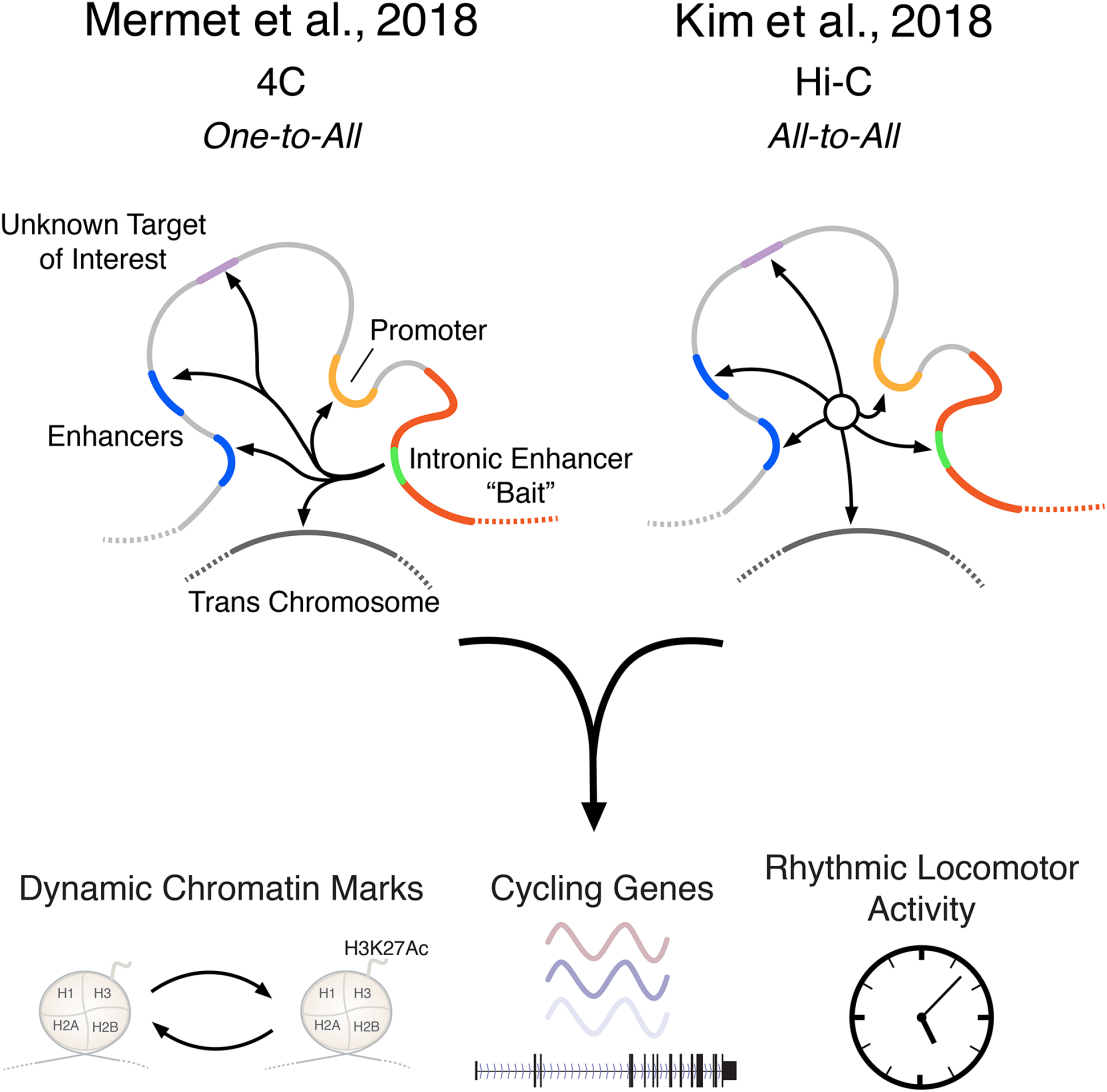
Adaptations of 3C technologies in two key studies link daily changes in genome topology to molecular and behavioral circadian rhythms. In this issue of *Genes & Development*, Mermet et al. (2018) used 4C-seq (circular chromosome conformation capture [4C] combined with sequencing) to demonstrate that enhancer–promoter loops play an essential role in rhythmic transcription and daily activity behavior. Concurrent work from Kim et al. (2018) used unbiased Hi-C (chromosome capture followed by high-throughput sequencing) technologies to identify rhythmic enhancer–promoter loops across the genome and the chromatin factors coordinating these rhythms. Together, these studies highlight the circadian dynamics of spatial genomic regulation.

Mermet et al. (2018) used circular chromosome conformation capture (4C), in which a target regulatory region is used as the “bait,” ligated contacts are circularized, and subsequent identification of bait contacts is performed by sequencing. One such bait used here was an established intragenic regulatory sequence within the gene encoding the clock repressor CRY1, previously identified in elegant work by Ukai-Tadenuma et al. (2011) as a modulator of rhythmic circadian oscillation. Contacts with the *Cry1* intragenic enhancer from nearby regions peaked during the subjective night (near the zenith of *Cry1* expression) and were diminished during the subjective day (the *Cry1* nadir). Of note, these patterns were present in both the kidney and the liver, a finding consistent with the observation that enhancer regulation of core clock loci is conserved across tissues (Perelis et al. 2015). In contrast, rhythmic intragenic promoter interactions were observed only in the liver for the gene encoding *glycogen synthase 2*, a clock output gene involved in glucose production, suggesting tissue specificity. Consistent with a role of the core circadian mechanism in driving *Cry1* enhancer–promoter looping, 4C-seq (4C combined with sequencing) analyses in *Bmal1* knockout mice displayed static elevated levels of *Cry1*. Since *Bmal1* mutant animals are also deficient in the circadian repressors REV-ERBα and REV-ERBβ and have elevated levels of the activator RORγ, one possibility may be that the loss of repression leads to static chromatin assembly. Enhancer–promoter loops thereby establish a transcriptional feedback loop!

Following the concept that functional experiments are necessary to discern the meaning of specific interactions captured by 4C-seq, Mermet et al. (2018) generated a transgenic mouse harboring a 300-base-pair deletion within the intronic *Cry1* enhancer. Animals bred to homozygosity with the loss of this single *Cry1* enhancer display significantly shortened period length under constant conditions, a hallmark of circadian clock disruption and consistent with the shortening observed in *Cry1* nullizygous mice, demonstrating the necessity of feedback repression in the core clock (Sato et al. 2006). As predicted, rhythmic chromatin contacts between the enhancer and the promoter were also lost in the enhancer mutant mice. At the cellular level, single-molecule RNA fluorescence in situ hybridization (smRNA-FISH) further corroborated a requirement for enhancer–promoter looping in de novo transcription. Collectively, the findings of Mermet et al. (2018) begin to uncover a new level of molding in the emergence of an intact rhythmic organism. The aforementioned work by Mermet et al. (2018) in intact animals also resonates with previous cell-based assays using 4C followed by microarray chip hybridization to identify distant regions interacting with an intragenic regulatory motif within the gene encoding the D-albumin-binding protein (DBP), a clock output factor (Aguilar-Arnal et al. 2013).

Technologies for detecting long-range chromatin interactions continue to evolve, enabling exploration of genome topology across all possible regulatory enhancer–promoter loops. In the “all-to-all” approach, chromosome capture followed by high-throughput sequencing (Hi-C) allows for the generation of unbiased contact maps. With sufficiently deep sequencing (as performed by Kim et al. 2018), topologically associated domains (TADs) and smaller localized sub-TADs give unbiased information on proximal DNA ligations and the frequency of such contacts. This provides a way in which *cis* and *trans* chromosome interactions are quantified at a resolution sufficient for mapping individual sites of exon/intron, enhancer, and promoter contacts. Using Hi-C, Lazar and colleagues (Kim et al. 2018) analyzed the mechanisms governing enhancer–promoter looping in proximate interactions under ∼300 kb. This “all-to-all” approach enabled unbiased identification of “time of day”-dependent interactions, with more observed during the subjective daytime, closer to the zenith of transcriptional activity of forward limb clock activators. Here, the studies also revealed correspondence between rhythmic chromatin interactions and rhythmic transcription, suggesting that topological transitions characterize rhythmic control of clock-controlled gene outputs. Intriguingly and echoing the findings of Mermet et al. (2018), *Cry1* enhancer–promoter looping was localized to a “time of day”-dependent region that emerged primarily during the subjective night. During the daytime, when expression of *Cry1* is at its nadir, directed analysis further confirmed REV-ERBα binding to the *Cry1* regulatory motif in an anti-phasic pattern with maximal expression of the gene, and liver-specific overexpression of REV-ERBα was sufficient to abrogate rhythmic *Cry1* expression and enhancer–promoter looping. Genetic analyses of enhancer–promoter looping in the absence of REV-ERBα implicate a direct role for this factor in both core clock and clock output regulation. Indeed, the activity of REV-ERBα in modulating enhancer–promoter looping appears to involve the recruitment of corepressors containing nuclear receptor corepressor and histone deacetylase 3. These modulate histone 27 Lys27 acetylation and corresponding docking of the transcriptional elongation factor bromodomain-containing protein 4 (BRD4) and the looping factor Mediator complex 1 subunit, leading to the induction of RNA polymerase II. Collectively, the work of Kim et al. (2018) places REV-ERBα as an integral component of core circadian gene regulation. The techniques used here pave the way for future functional genomic studies to address how each component of the core clock and clock-driven processes mediate circadian dynamics and the diverse cellular physiologic events each day.

Chromatin conformation analyses now provide a new view of core clock regulation and highlight gaps in our understanding of how the circadian system prepares organisms for both anticipated and unexpected changes in the environment. Importantly, circadian transcriptional and epigenetic signatures are shared across tissues, yet unique tissue-specific programs are required for organismal homeostasis (Perelis et al. 2015). Application of chromatin analyses to examine the extent to which core clock transcription factors and/or clock-recruited factors establish genomic topologies at the right time of day to impact physiology still remains an open area. Furthermore, mounting evidence suggests that core circadian processes exhibit dynamic responses to changes in the environment, raising the intriguing possibility that environmental signals might alter circadian chromatin programming. It appears that a new geometry is emerging, through which we can now view the shape of time.

## References

[GAD314187WEIC1] Aguilar-Arnal L, Hakim O, Patel VR, Baldi P, Hager GL, Sassone-Corsi P. 2013 Cycles in spatial and temporal chromosomal organization driven by the circadian clock. Nat Struct Mol Biol 20: 1206–1213. 24056944 10.1038/nsmb.2667 PMC3885543

[GAD314187WEIC2] Denker A, de Laat W. 2016 The second decade of 3C technologies: detailed insights into nuclear organization. Genes Dev 30: 1357–1382. 27340173 10.1101/gad.281964.116 PMC4926860

[GAD314187WEIC3] Fang B, Everett LJ, Jager J, Briggs E, Armour SM, Feng D, Roy A, Gerhart-Hines Z, Sun Z, Lazar MA. 2014 Circadian enhancers coordinate multiple phases of rhythmic gene transcription in vivo. Cell 159: 1140–1152. 25416951 10.1016/j.cell.2014.10.022 PMC4243056

[GAD314187WEIC4] Hardin PE, Hall JC, Rosbash M. 1990 Feedback of the *Drosophila* period gene product on circadian cycling of its messenger RNA levels. Nature 343: 536–540. 2105471 10.1038/343536a0

[GAD314187WEIC5] Kim YH, Marhon SA, Zhang Y, Steger DJ, Won KJ, Lazar MA. 2018 Rev-erbα dynamically modulates chromatin looping to control circadian gene transcription. Science 10.1126/science.aao6891. PMC5995144 29439026

[GAD314187WEIC6] Koike N, Yoo SH, Huang HC, Kumar V, Lee C, Kim TK, Takahashi JS. 2012 Transcriptional architecture and chromatin landscape of the core circadian clock in mammals. Science 338: 349–354. 22936566 10.1126/science.1226339 PMC3694775

[GAD314187WEIC7] Mermet J, Yeung J, Hurni C, Mauvoisin D, Gustafson K, Jouffe C, Nicolas D, Emmenegger Y, Gobet C, Franken P, 2018 Clock-dependent chromatin topology modulates circadian transcription and behavior. Genes Dev (this issue) 10.1101/gad.312397.118. PMC5900709 29572261

[GAD314187WEIC8] Perelis M, Marcheva B, Ramsey KM, Schipma MJ, Hutchison AL, Taguchi A, Peek CB, Hong H, Huang W, Omura C, 2015 Pancreatic β cell enhancers regulate rhythmic transcription of genes controlling insulin secretion. Science 350: aac4250. 26542580 10.1126/science.aac4250 PMC4669216

[GAD314187WEIC9] Sato TK, Yamada RG, Ukai H, Baggs JE, Miraglia LJ, Kobayashi TJ, Welsh DK, Kay SA, Ueda HR, Hogenesch JB. 2006 Feedback repression is required for mammalian circadian clock function. Nat Genet 38: 312–319. 16474406 10.1038/ng1745 PMC1994933

[GAD314187WEIC10] Ukai-Tadenuma M, Yamada RG, Xu H, Ripperger JA, Liu AC, Ueda HR. 2011 Delay in feedback repression by cryptochrome 1 is required for circadian clock function. Cell 144: 268–281. 21236481 10.1016/j.cell.2010.12.019

